# Coevolution in human small Heat Shock Protein 1 is promoted by interactions between the Alpha-Crystallin domain and the disordered regions

**DOI:** 10.1371/journal.pone.0321163

**Published:** 2025-05-05

**Authors:** Vanesa Racigh, Luciana Rodriguez Sawicki, Facundo Nicolas Eric Bravo, Maria Silvina Fornasari

**Affiliations:** 1 Departamento de Ciencia y Tecnología, Universidad Nacional de Quilmes, Bernal, Argentina; 2 Instituto de Investigaciones Bioquímicas de La Plata, CONICET-UNLP, Facultad de Ciencias Médicas, La Plata, Argentina; University of Toronto, CANADA

## Abstract

Human small Heat Shock Protein 1 (HSPB1) belongs to the Small Heat Shock Protein (sHSP) superfamily, a group of ATP-independent molecular chaperones essential for cellular stress responses and protein quality control. These proteins share a conserved domain organization, with a structured Alpha-Crystallin domain (ACD) flanked by disordered N-terminal and C-terminal regions (NTR and CTR). While the prevailing evolutionary hypothesis for the sHSP family suggests that the disordered regions evolved independently and at a faster rate than the ACD, this study provides, for the first time, evidence of coevolution between these regions in human HSPB1, introducing new insights into the evolutionary mechanisms that sustain critical regulatory interactions. By integrating evolutionary and structural approaches, we estimated evolutionary rates per region and position, analyzed the composition of key interacting motifs, and employed structural modeling with AlphaFold 2 to assess the prevalence of these interactions. Our findings reveal that while the disordered regions globally evolve faster than the ACD, specific motifs involved in regulatory interactions exhibit lower-than-average evolutionary rates, reflecting evolutionary constraints imposed by their functional importance. This coevolutionary mechanism may also extend to other small Heat Shock Proteins featuring interacting motifs in the NTR, CTR, or both, offering a new perspective for studying their molecular evolution. Furthermore, the analysis presented in this work could be applied to assess coevolution in other proteins with intrinsically disordered regions.

## Introduction

Small Heat Shock Proteins (sHSPs) are ATP-independent molecular chaperones that act as the first line of defense in the cellular chaperone network [1]. The human genome encodes ten sHSPs (HSPB1 to HSPB10), which can be ubiquitously expressed or exhibit tissue-specific expression patterns and become upregulated under conditions of cellular stress. Their primary role is to interact with misfolded clients to prevent aggregation. Variants of these proteins with altered chaperone activity are associated with several diseases, including Parkinson’s, Alzheimer’s, and neuropathies [2].

Structurally, sHSPs consist of a conserved Alpha-Crystallin domain (ACD) flanked by disordered N-terminal (NTR) and C-terminal (CTR) regions [3]. Depending on the subtype, sHSPs can assemble into dynamic homo- and hetero-oligomers of varying sizes, stabilized by extensive contacts between the disordered regions and the ACD [1,4,5]. In human HSPB1 and HSPB5, under stress conditions, specific phosphorylation sites in the NTR trigger the disassembly of oligomers into smaller species, freeing the NTR from oligomeric contacts and enhancing its interaction with exposed hydrophobic regions of misfolded proteins [6–8]. Experimental studies indicate that the dimeric form of these sHSPs exhibits the highest chaperone activity, making dimer constructs the standard unit for assessing chaperone function [8,9]. Each ACD dimer presents three grooves: a central groove at the dimer interface and two lateral grooves, one on each subunit. These grooves serve as interaction sites for specific motifs within the disordered regions, such as the I/V-X-I/V motif in the CTR and the conserved SRLFDQXFG motif in the NTR [3,10,11]. In higher-order assemblies, the CTR often functions as a non-covalent cross-linker between dimers by binding the I/V-X-I/V motif into the lateral groove of a neighboring dimer [5,12,13].

**Fig 1:**
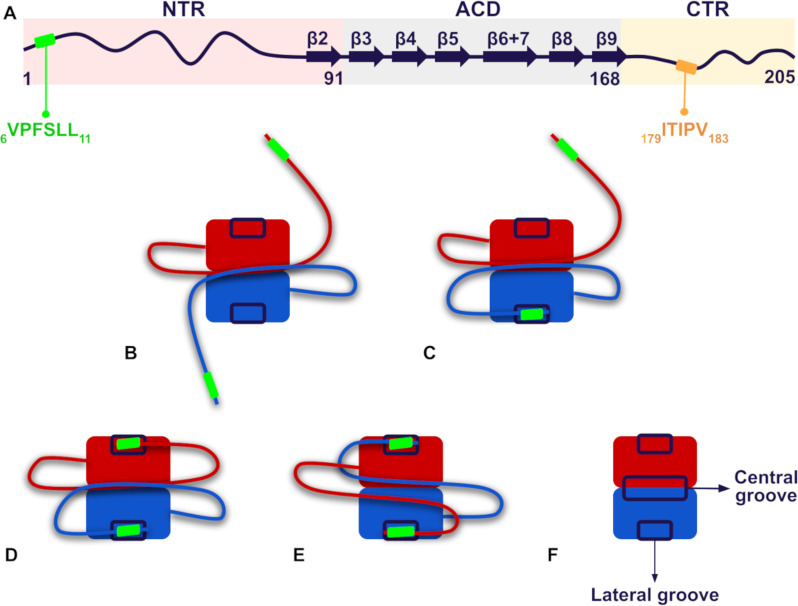
Schematic representation of human HSPB1 structure and the interactions between the distal segment of the NTR and the lateral grooves of the ACD via the _6_VPFSLL_11_ motif in the dimer, as described by Clouser et al. [ **14].** **A.** Primary and secondary structure of human HSPB1, indicating NTR, ACD, and CTR regions, as well as the _6_VPFSLL_11_ and _179_ITIPV_183_ motifs. **B-E.** The figures illustrate five possible interaction modes of the NTR’s distal region within the human HSPB1 dimer: **B.** both NTRs are free in solution; **C.** the NTR of one chain interacts with the lateral groove of the same chain while the NTR of the other subunit remains free; **D.** both NTRs are bound to the lateral grooves of their respective chain; and **E.** the NTR of each chain is in contact with the lateral groove of the opposite chain. In all modes, the CTR is omitted from the representation, while the global conformation of the NTR is schematically represented, with the _6_VPFSLL_11_ motif shown as a green rectangle. **F.** Lateral grooves that interact with the distal segment in the ACD dimer and the central groove that contacts other NTR regions.

Recent work by Clouser et al. provides a detailed experimental description of the interactions between the NTR and the ACD of human HSPB1 [14]. They found, following the canonical human HSPB1 sequence numbering, that the _6_VPFSLL_11_ motif located at the NTR interacts with the lateral grooves in a dimeric construct (Fig 1). Interestingly, experimental results indicate that abolishing this interaction enhances in vitro human HSPB1 chaperone activity towards its natural client tau [15]. Additionally, the crystal structure of the oligomeric form of human HSPB1 reveals that an overlapping or extended I/V-X-I/V motif (_179_ITIPV_183_) within the CTR also interacts with the ACD’s lateral grooves [5]. Moreover, the substitutions P7R, P7S, P182A, and P182L within the _6_VPFSLL_11_ and _179_ITIPV_183_ motifs are associated to Charcot-Marie-Tooth disease [16–21].

These findings underscore the crucial role of the interactions between the _6_VPFSLL_11_ and the _179_ITIPV_183_ motifs in the disordered regions with the ACD in the self-regulation of human HSPB1 chaperone activity. Both oligomer formation and the reversible interactions between the NTR and the ACD at the dimeric level are fundamental to this regulatory process. Given the current evolutionary hypothesis suggesting that the disordered regions of sHSPs evolved independently and at a faster rate than the ACD [22], it is relevant to explore how these contacts have shaped the evolution of human HSPB1. To investigate the evolutionary importance of these interactions, we used a manually curated dataset of HSPB1 orthologs to estimate the global evolutionary rates for the NTR, ACD, and CTR and to calculate site-specific evolutionary rates by taking the human HSPB1 sequence as the reference. Furthermore, we derived evolutionary insights from models generated with AlphaFold 2 (AF2) [23]. Our results suggest that the ACD, NTR, and CTR of human HSPB1 did not evolve independently; instead, they must have coevolved to maintain the critical interactions necessary for regulating its chaperone activity.

## Results

### HSPB1 ortholog dataset characterization

The curated dataset comprises 474 protein sequences, 199 from invertebrates and 275 from vertebrates. No sequences from other kingdoms, including Plantae, Fungi, Eubacteria, and Protista, remained in the dataset after curation and filtering. Among vertebrates, the dataset includes 88 mammals, 124 fish, 26 birds, 26 reptiles, and 11 amphibians. The multiple sequence alignment (MSA) analysis shows that the length of ACD is highly uniform, with an average of 77.0 ±  0.5 amino acids. The NTR is longer than the CTR (88.8 ±  18.3 and 34.7 ±  8.4 respectively). The CTR lengths are similar in vertebrates (32.1 ±  8.1) and invertebrates (38.2 ±  7.5). In contrast, the NTR length shows more variability between vertebrates (99.5 ±  16.6) and invertebrates (74.0 ±  6.5). This difference is due to a low-complexity region of variable length present only in vertebrates, known as the inserted segment in human HSPB1 [14].

The analysis reveals a high conservation of ACD composition across all organisms. S1 Fig shows that the percentage of each type of amino acid in the ACD is similar for most amino acids in the vertebrate and invertebrate sets. The NTR contains a higher proportion of aromatic amino acids (TRP, TYR, and PHE), which are nearly absent in the CTR; this enrichment, although unusual for intrinsically disordered regions, is commonly associated with molecular recognition regions that facilitate protein-protein interactions [24,25].

The amino acid composition of the NTR is more variable between vertebrate and invertebrate sets, with a notable presence of hydrophobic and positively charged amino acids, especially in vertebrates. In contrast, the CTR exhibits a more uniform composition across different organism sets. It contains a higher proportion of polar and negatively charged amino acids, which may help maintain the protein in solution in the dimeric context. Although both regions are disordered, they exhibit distinct compositions linked to their specific roles. Regarding the structured domain, human HSPB1 contains a single cysteine residue in the ACD (C137), which forms a disulfide bridge upon oxidation and helps to keep the dimer bonded, as shown by experimental data (PDB structure 2N3J) [26]. Cysteines form disulfide bonds that can influence the oligomeric equilibrium of sHSPs. Thus, it is intriguing that some organisms have cysteines in the NTR and the CTR in addition to the ACD, despite their low proportion.

### NTR and CTR motifs that interact with the ACD are conserved across all HSPB1 orthologs

We further explored the composition of the _6_VPFSLL_11_ motif in the NTR and the _179_ITIPV_183_ motif in the CTR. S1 Table shows compositional data. Both motifs show distinct patterns between vertebrates and invertebrates. An essential aspect of this analysis is the consideration of proline 7 in the NTR, fully conserved across both vertebrates and invertebrates, as a reference point for defining the position of the _6_VPFSLL_11_ motif in invertebrates. In vertebrates, the _6_VPFSLL_11_ motif occurs in 27.6% of sequences, while the three most frequent alternatives, VPFSLL, VPFTFL, and IPFTLL, account for 62.9% of sequences.

Among vertebrates, the _179_ITIPV_183_ motif in the CTR is highly conserved. Alternatives of the I/V-X-I/V motif are present in 98.5% of the recruited sequences, while the extended form ITIPV is predominant, found in 56.0% of sequences. Notably, the non-extended form TTIPV occurs in 20.4% of sequences and is observed exclusively in fish species. In contrast, invertebrates display only non-extended forms of this motif. Proline 182 exhibits conservation in 87.5% of total sequences.

Analysis of the lateral grooves formed by the β4 and β8 strands within the ACD (positions _109_LTVKT_113_ and _153_VSSSL_157_) highlights significant conservation of key amino acids. In the β4 strand of vertebrates, leucine, valine, and lysine are the predominant amino acids, while in the β8 strand, valine, serine, and leucine are the most frequent ones. Although invertebrates show a more variable composition of the β4 and β8 strands, the residues primarily vary by others with chemically equivalent or similar side chains, suggesting preservation in the structural characteristics of the lateral grooves. This analysis indicates that the composition of the lateral grooves is highly conserved, and despite being located in disordered regions, the motifs interacting with the ACD are also highly conserved or exhibit variants that may fulfill roles similar to those of the _6_VPFSLL_11_ and _179_ITIPV_183_ motifs in human HSPB1. Therefore, the interactions relevant to the self-regulation of human HSPB1 might be characteristic of all HSPB1 orthologs.

### Disordered regions show faster evolutionary rates than conserved ACD

As mentioned above, the current evolutionary hypothesis for the sHSP family suggests that the disordered regions evolved independently and at a faster rate than the ACD [22]. To assess this hypothesis, we divided sequences in the complete dataset into NTR, CTR, and ACD segments to create three subsets. These were employed to calculate the evolutionary distances for each pair of organisms. The underlying assumption behind this protocol is that the evolutionary time elapsed between each pair of organisms is equivalent across the three subsets, as they derive from the complete set of orthologous sequences. Consequently, if the entire protein were under the same evolutionary constraints, a similar distribution of evolutionary distances would be expected for the three regions.

**Fig 2:**
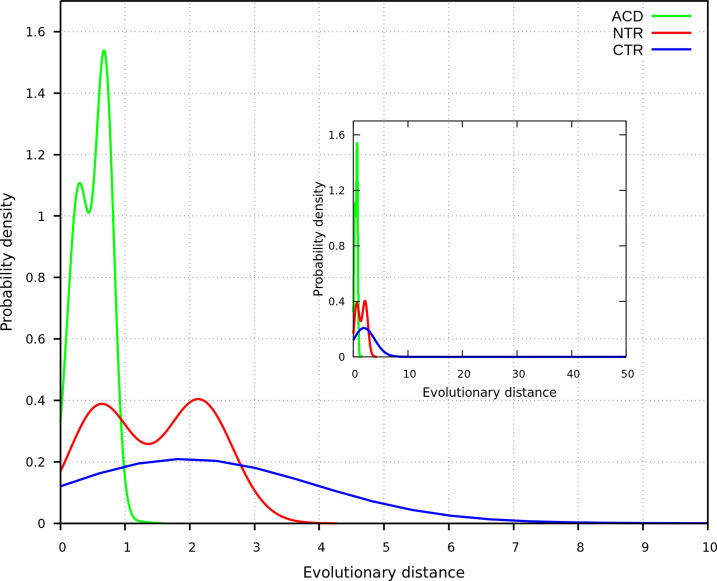
Probability densities of evolutionary distances for the disordered regions and the ACD. The inset displays the full range of distance values.

As shown in Fig 2, probability density functions calculated for the evolutionary distances of the NTR, CTR, and ACD display distinct patterns. This observation indicates that these regions have evolved at different rates. The distribution for the ACD (green curve) exhibits a sharp peak at low evolutionary distances, likely due to the structural constraints limiting the variability of the ACD. In contrast, the distributions for the NTR and CTR are broader, suggesting that these disordered regions are less constrained and have accumulated more sequence changes over time. Furthermore, the NTR distribution is slightly shifted towards lower distances relative to the CTR, implying that this region may be under stronger evolutionary pressure.

Moreover, the NTR distribution appears bimodal corresponding the first peak to intra-group comparisons within vertebrates and invertebrates. It is associated with shorter evolutionary distances, suggesting recent divergence within each group. The second peak, arising from comparisons between vertebrate and invertebrate sequences, reflects higher evolutionary distances and more divergence between these lineages. A similar pattern is observed in the ACD curve, though the distance between the peaks is smaller, highlighting the higher conservation of this domain across species. The inset within Fig 2 shows the full range of evolutionary distances. Distributions are centered within a limited range, although some sequences in the CTR encompass significantly higher distances, reflecting more extensive divergence in that region.

The statistical analysis, as shown by the Kolmogorov-Smirnov (KS) test, supports these observations. The KS statistic values for the comparisons between NTR and ACD, CTR and ACD, and NTR and CTR are 0.60, 0.80, and 0.31, respectively, with p-values < 1e-16 for all comparisons. These results confirm significant differences in the evolutionary rates of these regions. The broader and more right-shifted probability density distributions for the NTR and CTR, compared to the ACD, imply that these regions have accumulated more evolutionary changes. Specifically, the higher evolutionary distances observed for the CTR imply that it has experienced the fastest rate of evolution, followed by the NTR, with the ACD being the slowest.

### Motifs in disordered regions interacting with the ACD show reduced evolutionary rates

**Fig 3:**
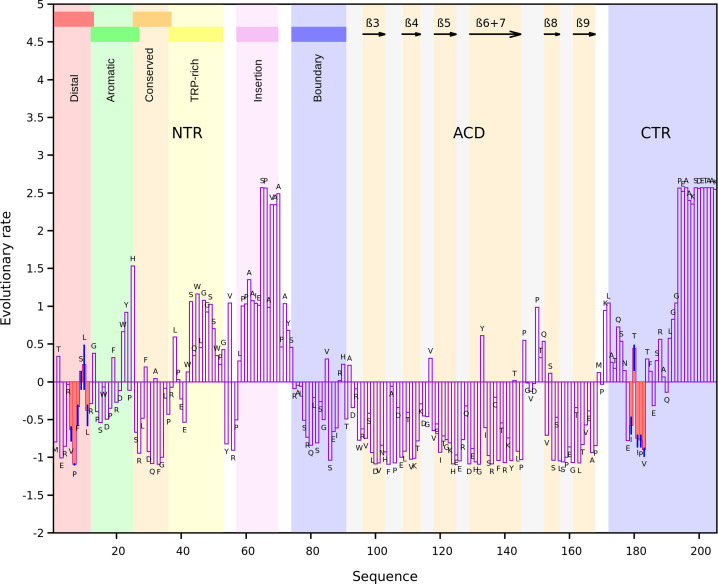
Per position evolutionary rate profile for the canonical human HSPB1 sequence estimated using the full ortholog dataset. The background highlights the NTR, ACD, and CTR. The NTR consists of six segments [14]: distal (residues 1-13), aromatic (residues 12-27), conserved (residues 25-37), Trp-rich (residues 37 to 53), inserted (residues 57-70), and boundary (residues 74-91). Black arrows indicate the β strands of the ACD. Red highlights the _6_VPFSLL_11_ motif of the NTR and the _179_ITIPV_183_ motif of the CTR. The QQ intervals, shown in blue for these motifs, represent the interquartile range (P25–P75) of the estimated evolutionary rates, capturing the range within which the central 50% of values fall, providing insight into their variability.

In light of the previous results, we estimated the evolutionary rates per site of the human HSPB1 sequence using the complete dataset. The profile obtained (Fig 3) reveals a clear distinction in the rates of evolution across different regions of the protein. Residues that constitute the ACD predominantly exhibit evolutionary rates lower than the average rate of the complete sequence, while the NTR and the CTR exhibit a more heterogeneous pattern.

**Fig 4:**
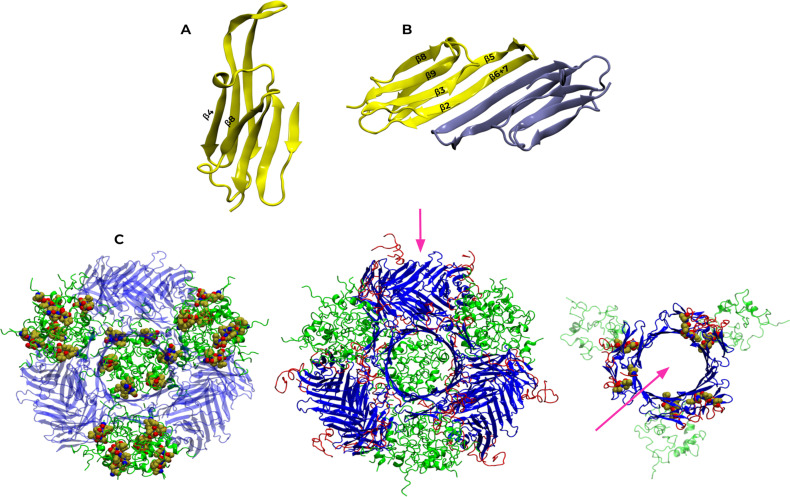
Structural overview of human HSPB1 highlighting the ACD and its oligomeric assembly. **A.** ACD structure of human HSPB1 (PDB ID 4MJH, chain A), showing β4 and β8 strands forming the lateral groove. **B.** Top view of the ACD dimer, indicating the positions of the β-strands. **C.** 24-subunit oligomeric structure of human HSPB1 (PDB ID 6DV5) displaying four circular arrangements of three dimeric ACDs (blue) held together through extensive interactions mediated by the NTRs (green). The CTRs of each subunit within the dimers (red) are in contact with the lateral grooves of neighboring subunits. The pink arrow indicates the perspective of the top view on the right, showing one of the four arrangements of six subunits that make up the oligomer. This structure highlights the _179_ITIPV_183_ motif of each subunit interacting with the lateral groove of the adjacent subunit. The left structure shows _6_VPFSLL_11_ residues within the NTRs arrangement in sphere representation.

Within the ACD, the β4 and β8 strands framing the lateral grooves (Fig 4A) show lower-than-average rates that may result from structural constraints to preserve the ACD structure and functional requirements, as these strands not only interact with motifs in the disordered regions but also with other proteins as it is the case of co-chaperone BAG3 and client proteins [3,27]. The CTR generally exhibits higher-than-average evolutionary rates, except in positions adjacent to and within the _179_ITIPV_183_ motif, whose rates fall below the average. Residues T180 and P182 that correspond to the variable position in the I/V-X-I/V motif exhibit opposite evolutionary rates: T180 has a high rate, whereas P182 shows a low rate. This may result from the absence of the extended motif form in invertebrates and fish, leading to T180’s lack of conservation compared to P182’s high prevalence across organisms.

In terms of NTR, it was divided into six segments (distal, aromatic, conserved, Trp-rich, inserted, and boundary) as defined by Clouser et al [14] (Fig 3). These authors used a phosphomimetic construct of human HSPB1 in their work. Hereafter, any reference to experimental results related to the dimeric form will pertain to this construct. Their study shows that four NTR segments interact with the ACD (distal, aromatic, conserved, and boundary). Interestingly, the evolutionary rates estimated for the corresponding positions show lower-than-average values despite being located in a disordered region. The distal segment, which contains the _6_VPFSLL_11_ motif, engages with the lateral grooves of the ACD. The positions within this motif exhibit evolutionary rates below the sequence average, except for residues _9_SL_10_, which show slightly higher values due to their variability in the alignment. However, the QQ interval encompasses both negative and slightly positive values, indicating fluctuations around the sequence average rate. The conserved segment includes the _26_SRLFDQXFG_34_ motif and interacts with the central groove. The aromatic segment also interacts with the ACD. In vertebrates, this region contains alternative sequences of a characteristic motif in the NTR of sHSPs, _16_WDPF_19_ [28], involved in chaperone oligomerization [29,30]. Additionally, the boundary segment adopts an antiparallel β-sheet conformation (β2) with the β3 strand of the ACD within the same chain (Fig 4.B) [14]. Thus, the reduced evolutionary rates observed for these segments could originate from a differential selective pressure due to the functional importance of their interactions with the ACD, contrary to the behavior observed for the Trp-Rich and the inserted segments. These do not interact consistently with the ACD in the dimeric form. In particular, the insertion segment behaves as a solvated random coil [14]. That might explain the higher rates observed for positions within these segments, as solvent-exposed regions tend to evolve more rapidly than those with lower accessible surface areas [31]. Also, the length and composition of the inserted segment vary significantly among vertebrates, contributing to the higher evolutionary rates observed in this region.

Analysis of the rate profile must consider both contacts at the dimeric level and those involved in large oligomer formation. Therefore, we mapped the interactions in the 24-subunit oligomeric structure of human HSPB1 (PDB structure 6DV5). This structure is an arrangement of four groups of three dimer pairs connected through extensive NTR interactions (Fig 4C). The inserted segment and part of the boundary segment were not solved due to their high mobility [5]. In this structure, the NTRs do not establish interchain interactions with the ACD (S2 Table). On the other hand, contacts between the CTR and the ACD represent 10.5% of the total interchain interactions in the oligomer (S2 Table). The CTR of each chain engages the lateral groove of the neighboring one, positioning the _179_ITIPV_183_ motif within its groove. Nevertheless, although the PDB structure constitutes a valuable source of information about the complex topology, it only captures a single conformation of the oligomer. Thus, the number and nature of the interactions in this structure might differ in a dynamic context.

Additional experimental evidence supports the significance of the interactions between the _6_VPFSLL_11_ and the _179_ITIPV_183_ motifs with the lateral grooves. Phosphomimetic mutations (S15D, S78D, and S82D) alone do not prevent oligomerization, as the _179_ITIPV_183_ motif still interacts with the lateral grooves [9], and substituting it with _179_GTGPG_183_ is essential to isolate a dimeric form. Moreover, structural data from all oligomeric sHSPs in the PDB show that the conserved I/V-X-I/V motif interacts with the ACD’s lateral grooves [11,32,33], while NTR arrangements vary significantly [1,33]. Oligomer polydispersity, diversity in NTR arrangements, variability in NTR length, and the lack of PDB structures for many sHSPs make it imprudent to assess _6_VPFSLL_11_ role in oligomerization based solely on the 24-mer structure. Notably, a human HSPB1 construct lacking residues 1-14 can still form oligomers [34], indicating oligomerization is not entirely dependent on this region. Conservation of the motif across orthologs and its role in regulating dimeric chaperone activity [15] suggest selective pressures likely constrain these positions to preserve this role.

### Structural modeling of human HSPB1 with AlphaFold 2 reveals insights on coevolution

Structural data is available for the NTR in the phosphomimetic dimer of human HSPB1 [14]. However, there is currently no available structure exhibiting the interaction between the _6_VPFSLL_11_ motif and the lateral grooves in the wild-type dimer. Therefore, we used AF2 to generate 500 structures of this dimer to investigate whether this interaction is predicted and to evaluate the frequency of this contact across the models. We used this approach because, unlike our manually curated dataset of 474 orthologous sequences, AF2 does not limit its MSA to strict orthologs. Instead, it automatically recruits homologous sequences, including both orthologs and paralogs, using JackHMMER [35] and HHblits [36] to search UniRef90, BFD, and MGnify. Through this process, AF2 initially retrieved 10019 sequences but applied an internal filtering mechanism to reduce redundancy and maximize sequence diversity, capping the final MSA at 2048 sequences for computational efficiency [37]. This large-scale sequence sampling enables AF2 to leverage coevolutionary signals from a more divergent MSA, allowing us to assess the prevalence of the relevant interactions within this broader evolutionary context.

Analysis of the models reveals conservation in the structure of the ACD (residues 92 to 168) across all models. RMSD for backbone atoms was 0.51 ±  0.15, using as reference the crystal structure of a dimeric construct containing only the ACD of human HSPB1 (PDB ID 4MJH). On the contrary, the disordered regions adopt various conformations (S2 Fig). The average per residue estimate of confidence (pLDDT) exhibited values above 70 with low standard deviation for the residues forming the ACD, which indicates that this region is well modeled (S3 Fig). In particular, positions with values close to or exceeding 90 suggest high model accuracy. In contrast, pLDDT scores for the residues within the CTR were below 50, as expected due to their disordered nature [38]. On the other hand, the high pLDDT scores for the distal segment of the NTR containing the _6_VPFSLL_11_ motif align with AF2’s capacity to identify conditionally folded intrinsically disordered regions. This interaction-driven structural stabilization of the NTR likely reflects the coevolutionary signals captured in the MSA used by AF2 [39].

**Fig 5:**
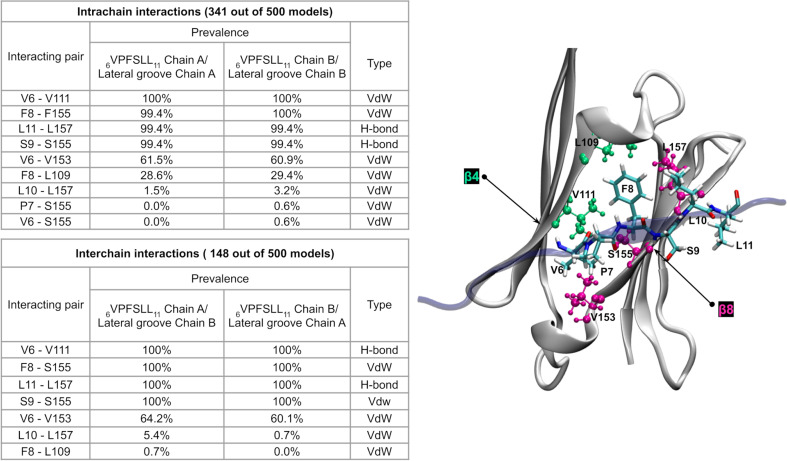
Summary of intrachain and interchain interactions between the _6_VPFSLL_11_ motif and the lateral grooves of the ACD (β4 and β8 strands) in human HSPB1 dimer models. The prevalence of interactions is shown as a percentage of the total number of models in which each type of contact (intra or interchain) occurs, along with the nature of the contact (Van der Waals or H-bond). The right panel highlights the residues involved in the interactions in the table.

Using the interaction fingerprint obtained from the model set, we assessed whether the _6_VPFSLL_11_ motif was in contact with the lateral grooves of the ACD. We analyzed each chain separately, and the contacts present were symmetric between the two chains. Our results reveal that in 98.2% of the predicted structures, the _6_VPFSLL_11_ motif was in contact with one of the lateral grooves of the dimer through Van der Waals and H-bond interactions (Fig 5). In the remaining 1.8%, the NTR was not in contact with the lateral grooves but instead adopted an extended conformation. None of the models showed the _179_ITIPV_183_ motif of the CTR interacting with the grooves. For the _6_VPFSLL_11_ motif, 68.6% of the interactions were intrachain contacts, while 29.6% were interchain contacts. Both types of interactions are consistent with the quasi-ordered states that human HSPB1 can adopt [14].

Further analysis to characterize residue pairs that constitute this interaction in the models reveals that the F8-S155 pair interacts consistently in nearly 100% of models displaying contacts, regardless of whether these interactions are intra or interchain. This observation aligns with findings by Baughman et al., who reported that substitutions at these specific positions (F8G and S155Q) in the dimer of human HSPB1 release its NTR enhancing its chaperone activity toward the natural client tau [15]. In a complementary analysis, we generated 25 additional models with AF2 configured to rely exclusively on MSA data, to observe how AF2 infers contacts relying only on sequence-derived evolutionary signals. During recycling, AF2 generates distograms as intermediate outputs, which predict the probabilistic distances between residue pairs and help refine structural predictions. Although these distograms are not final outputs, they provide insight into the algorithm assessment of inter-residue relationships. In these template-free models, the ACD structure of the dimers remained conserved.

**Fig 6:**
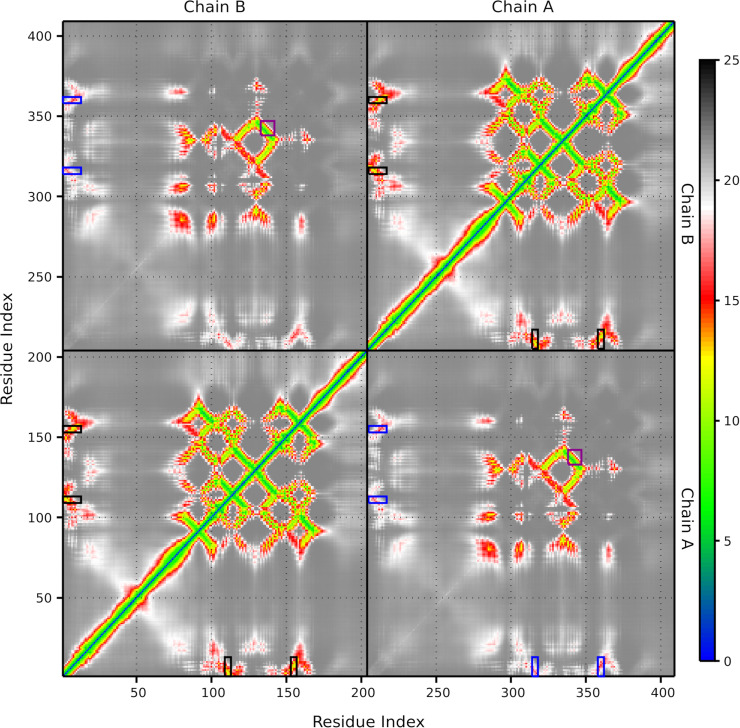
Average distogram generated from individual distograms produced by AF2 for human HSPB1 dimer models without including the PDB70 database. Colors represent the probabilistic distances between residue pairs, with blue indicating short distances and red indicating long distances. Black and blue rectangles highlight the intrachain and interchain contacts between the distal segment containing the _6_VPFSLL_11_ motif and the β4 and β8 strands that shape the lateral grooves. Purple rectangles emphasize the intersection of the β6/7 strands from each chain (residues 133-142). Dividing lines denote the areas corresponding to each chain, with residue index ranging from 1 to 205 for Chain A and 206 to 410 for Chain B.

The distogram shown in Fig 6 was generated by averaging the distograms from individual models, and standard deviations were calculated for each point. Residues 1-91, 92-168, and 169-205 of Chain A and their respective counterparts in Chain B (206-410) represent the NTR, ACD, and CTR, respectively. The ACD forms the central block, with shorter intrachain distances (~5 Å) shown in green, reflecting conserved interactions within each ACD. The purple rectangles highlight the β6/7 strands (133-142), forming interchain contacts around ~ 5 Å, consistent with the structured and conserved nature of the ACD dimer interface. Residues belonging to the CTR do not appear to establish contacts with the ACD. In contrast, the distal segment of the NTR of each chain (residues 1 to 13) comprising the _6_VPFSLL_11_ motif and the β4 and β8 strands (residues 109 to 113 and 153 to 157) are predicted to be in contact. Intrachain contacts between this segment and each strand are marked by black rectangles, with distances ranging from ~ 10 Å to 15 Å. Interchain contacts, highlighted in blue, show distances of ~ 15 Å to 20 Å. These values suggest transient or flexible associations rather than direct atomic interactions. It is important to note that these distances are not final, as seen in the relaxed models used for evaluating contact occurrence with Prolif. The standard deviation for distances corresponding to the ACD remains close to 0, indicating consistent predictions; in contrast, the NTR-lateral groove contacts exhibit deviations up to 2.5 Å, reflecting greater flexibility in these regions (data not shown).

Notably, even in this template-free setup, where AF2 utilized a more divergent MSA that includes paralogous sequences, the interaction between the NTR and the lateral groove was still predicted. This suggests that these contacts are strongly encoded in the evolutionary sequence information, regardless of whether the MSA is restricted to orthologs or includes a broader set of homologs.

## Discussion

In this work, we explore the implications of the interactions between the _6_VPFSLL_11_ motif of the NTR and the _179_ITIPV_183_ motif of the CTR with the ACD in the evolution of human HSPB1. These contacts play a key role in its chaperone activity self-regulation: the _6_VPFSLL_11_ motif has a regulatory role at the dimeric level, while the _179_ITIPV_183_ motif contributes to oligomer formation [14,15,40]. The current evolutionary hypothesis for the sHSPs superfamily suggests that the NTR and CTR evolved independently and at different rates than the conserved ACD [22]. Since residues involved in functionally relevant interactions are often evolutionarily constrained and tend to coevolve to maintain their roles [41], we conducted evolutionary analysis and structural modeling to assess coevolution between the disordered regions and the ACD of human HSPB1.

Therefore, we worked with a manually curated set of orthologous sequences of human HSPB1. This dataset revealed that the amino acid composition of the ACD is conserved in both vertebrates and invertebrates, while that of the NTR and CTR exhibit higher variability. MSA analyses indicate that the composition of the lateral grooves of the ACD is highly conserved. Furthermore, the positions of the _6_VPFSLL_11_ and _179_ITIPV_183_ motifs are either preserved in orthologs or replaced by amino acids with comparable physicochemical properties, likely maintaining their functional interaction with the ACD. This result is consistent with many motif-binding domains exhibiting weak specificity, interacting primarily with a small core of residues while tolerating substitutions that retain essential binding characteristics, thereby allowing critical interactions to persist despite evolutionary divergence [42,43].

Furthermore, we replicated the methodology used by Kriehuber et al. in their work to assess whether our dataset mirrors the evolutionary pattern they reported [22], even though it contains fewer divergent sequences. Assuming uniform evolutionary constraints would produce similar rates across all regions, our findings show distinct rates: the ACD evolves the slowest, followed by the NTR, with the CTR evolving the fastest. Although disordered regions collectively evolve faster than the ACD, individual positions do not evolve uniformly. In particular, sites involved in ACD interactions, such as the _6_VPFSLL_11_ and _179_ITIPV_183_ motifs, evolve more slowly than the average of the complete sequence, likely due to selective pressures preserving these critical interactions. This result explains why the NTR, which is longer than the CTR, has a lower overall evolutionary rate, as it contains an increased number of ACD-interacting motifs.

Furthermore, models of human HSPB1 dimer generated with AF2 capture the interaction between the _6_VPFSLL_11_ motif and the lateral grooves of the ACD according to the quasi-ordered states described for this protein [14]. Notably, models generated without templates also predict these contacts. This consistency indicates that the coevolutionary-like relationships inferred by AF2 are grounded in evolutionary constraints, supporting their use as evidence of coevolution in our analyses.

Altogether, this evidence suggests that, although the disordered regions of HSPB1 exhibit higher substitution rates on average compared to the ACD, they have not evolved independently; instead, they have likely coevolved with the ACD to preserve essential interactions necessary for chaperone activity regulation. These findings align with studies on proteins with disordered regions, which often show evolutionary conservation at positions or motifs within these regions that interact with ordered domains [1,44,45].

Moreover, this evolutionary perspective provides a context for understanding why substitutions in conserved motifs within disordered regions, such as P7S and P7R variants in the _6_VPFSLL_11_ motif or the P182A and P182L variants in the _179_ITIPV_183_ motif, are associated with Charcot-Marie-Tooth disease [16,21]. These substitutions likely alter the motif-ACD interaction, potentially affecting human HSPB1 chaperone function, as even a single amino acid change at a critical site can be enough to disrupt binding [43]. In this line, a study shows that P182, while not directly interacting with the ACD, restricts conformational flexibility in the _181_IPV_183_ motif, facilitating its interaction with the lateral grooves. Substituting proline with leucine increases the motif’s flexibility, reducing its binding affinity to the ACD [16].

Interactions between specific motifs from the disordered regions (I/V-X-I/V variants in the NTR and CTR) occur in other human paralogs [13,46,47]. Competition for the lateral grooves between these motifs has been reported for human HSPB5 [48], similar to the interactions seen with _6_VPFSLL_11_ and _179_ITIPV_183_ described by Clouser et al. [14]. Given that various human paralogs feature interaction motifs in the NTR, CTR, or both [3], it is plausible that coevolutionary processes driven by selective pressures to maintain critical interactions could also occur in other members of the sHSP family. Further studies integrating structural and evolutionary information with the impact of disease-associated variants on chaperone activity could improve our understanding of the functional diversity within the sHSP family.

## Materials and methods

### HSPB1 ortholog dataset creation

Homologous protein sequences were initially recruited from the UniprotKB database [49] with Uniprot BLASTp [50], using the canonical human HSPB1 (UniProt ID P04792) as a query. To ensure a comprehensive dataset, additional sequences were sourced by filtering according to specific taxonomic groups. The recruitment process followed the phylogenetic tree provided by Hedges et al. [51], focusing on major kingdoms: Animalia, Plantae, Eubacteria, Fungi, and Protista. When the maximum number of sequences was reached for a kingdom, further recruitment was conducted within subgroups of that kingdom to capture broader diversity. After recruitment, an initial filtering step was applied to remove sequences that were partial, hypothetical, or contained indeterminate residues. Duplicated sequences were also eliminated to avoid redundancy. The remaining sequences were aligned and a threshold of 30% identity and 40% coverage was applied using an in-house program that implements Biopython [52]. The resulting dataset was manually curated to compile a set of orthologous HSPB1 sequences for subsequent analysis. The importance of working with proteins coded by orthologous genes is related to studying the evolutionary history of the product of a single gene, ensuring that these are the same protein in different organisms and, consequently, perform the same function.

### Dataset characterization

Two subsets were generated from the ortholog dataset, one for vertebrates and another for invertebrates. The three sets were aligned using Clustal Omega within UGENE [53]. The amino acid composition and the observed substitutions of the _6_VPFSLL_11_ motif within the NTR and the _179_ITIPV_183_ (I/V-X-I/V variant) in the CTR were analyzed from the alignments. Additionally, the amino acid composition of the β4 and β8 strands (positions _109_LTVKT_113_ and _153_VSSSL_157_) was examined.

### Structural and evolutionary analysis

The ortholog dataset was divided into three subsets, each corresponding to a region of the protein (NTR, ACD, and CTR). Each subset was subsequently aligned. Evolutionary distances within the NTR, CTR, and ACD datasets were calculated with the protdist program from the PHYLIP package with default parameters [54], following the protocol described by Kriehuber et al. [22]. To visualize the distribution of these evolutionary distances, we applied Kernel density estimation to the flattened distance matrices. Differences in evolutionary rates between these regions were statistically assessed through the Kolmogorov-Smirnov test implemented in Python. The evolutionary rate per site was calculated employing the Rate4Site program with default parameters [55]. The analysis was conducted on the HSPB1 ortholog dataset, with the canonical human HSPB1 sequence as the reference.

To assess the prevalence of the interaction between the _6_VPFSLL_11_ and _179_ITIPV_183_ motifs and the lateral grooves of human HSPB1, first, the intra and interchain interactions in the 24-mer oligomer of human HSPB1 (PDB ID 6DV5) were mapped with the RING web server [56]. Subsequently, structural models of the human HSPB1 dimer were generated with AF2 [23] on a local installation with A30 GPUs. The canonical sequence of human HSPB1 was used as input, along with the UniRef90, MGnify, and BFD sequence databases, and the PDB70 structural database. A total of 500 relaxed structures were generated (5 models with 100 predictions each) in PDB format. The files containing the structures were reformatted with pdb4amber into a PDB format compatible with the ProLIF toolkit [57] and stacked into a single file for processing with CPPTRAJ, which was also used to calculate the RMSD relative to the PDB structure 4MJH [46]. Both tools are part of the AmberTools package [58]. The combined file was then analyzed with the ProLIF toolkit to obtain an interaction fingerprint between the residues of the _6_VPFSLL_11_ and _179_ITIPV_183_ motifs and those forming the β4 (_109_LTVKT_113_) and β8 (_153_VSSSL_157_) strands. A contact was defined based on ProLIF’s detection of interactions between any residue in the motifs and any residue in the β4 or β8 strands. Per residue pLDDT values were extracted from the PDB files of the relaxed models using custom Python scripts. Structural models were visualized with VMD [59].

Next, to observe the contacts inferred by AF2 based solely on the MSA information, an additional set of 5 models of the HSPB1 dimer was generated, with 5 predictions per model (25 structures). Templates from the PDB70 database were not included in this prediction. The distance histograms (distograms) information generated by AF2 for each model were extracted from the.pkl files employing the dgram2dmap tool [60].

## Supporting information

S1 Fig Amino acid composition of the NTR, CTR, and ACD in the HSPB1 sequences from the full dataset, as well as the vertebrate and invertebrate subsets. (TIF)

S2 Fig Representative conformations of the disordered NTR and CTR in human HSPB1 models generated with AlphaFold 2. Models A and B depict interchain and intrachain interactions between the NTR and the ACD, with the CTR remaining in solution. Model C shows a conformation where neither the NTR nor the CTR interacts with the ACD. This modeling aimed to determine whether interactions between the _6_VPFSLL_11_ motif and the ACD could be captured, regardless of the specific conformations adopted by the rest of the NTR. Models D and E display two perspectives of the same conformation from a dimer model generated using HSPB1’s phosphomimetic sequence, modeled with ColabFold [61] and employing PDB 4MJH as a template. In the highest-ranked model among the five provided by the server, the distal (which adopts a β-sheet structure upon binding to the lateral grooves), aromatic, conserved, Trp-rich, inserted, and boundary segments adopt conformations consistent with the experimental description provided for each region by Clouser et al., while the CTR remains in solution.
(TIF)

S3 Fig Average pLDDT values for each residue across 500 models of the human HSPB1 dimer generated with AlphaFold 2. Residues 1 to 205 correspond to Chain A, and residues 206 to 410 correspond to Chain B. Background colors highlight the residue ranges for the NTR, ACD, and CTR. Green error bars indicate the standard deviation for key residues across different regions: P7 (from the _6_VPFSLL_11_ motif), F29 (from the conserved _26_SRLFDQXFG_34_ motif), L109 (β4 strand), V153 (β8 strand), and P182 (from the _179_ITIPV_183_ motif).
(TIF)

S1Table Per-position percentage composition of amino acids in the sequence alignment of vertebrates, invertebrates, and the full dataset for the VPFSLL, I/V-X-I/V motifs, and the β4 and β8 strands. For the motifs located in disordered regions, the three most frequent alternative motifs in each alignment are indicated. Only the percentage of the predominant amino acids (up to three) is shown in each position.
(DOCX)

S2 Table Percentage of intra and interchain interactions involving the NTR (residues 1–91), ACD (residues 92–168), and CTR (residues 169–205) in the 24-mer structure of human HSPB1 (PDB ID 6DV5). Values represent the proportion of interactions between and within each region relative to the total mapped interactions.
(DOCX)
