# Supplementary figures and images for: Coevolution in human small Heat Shock Protein 1 is promoted by interactions between the Alpha-Crystallin domain and the disordered regions
Source: PLoS One. 2025 May 5;20(5):e0321163. doi: 10.1371/journal.pone.0321163 (PMC12052118; doi:10.1371/journal.pone.0321163)

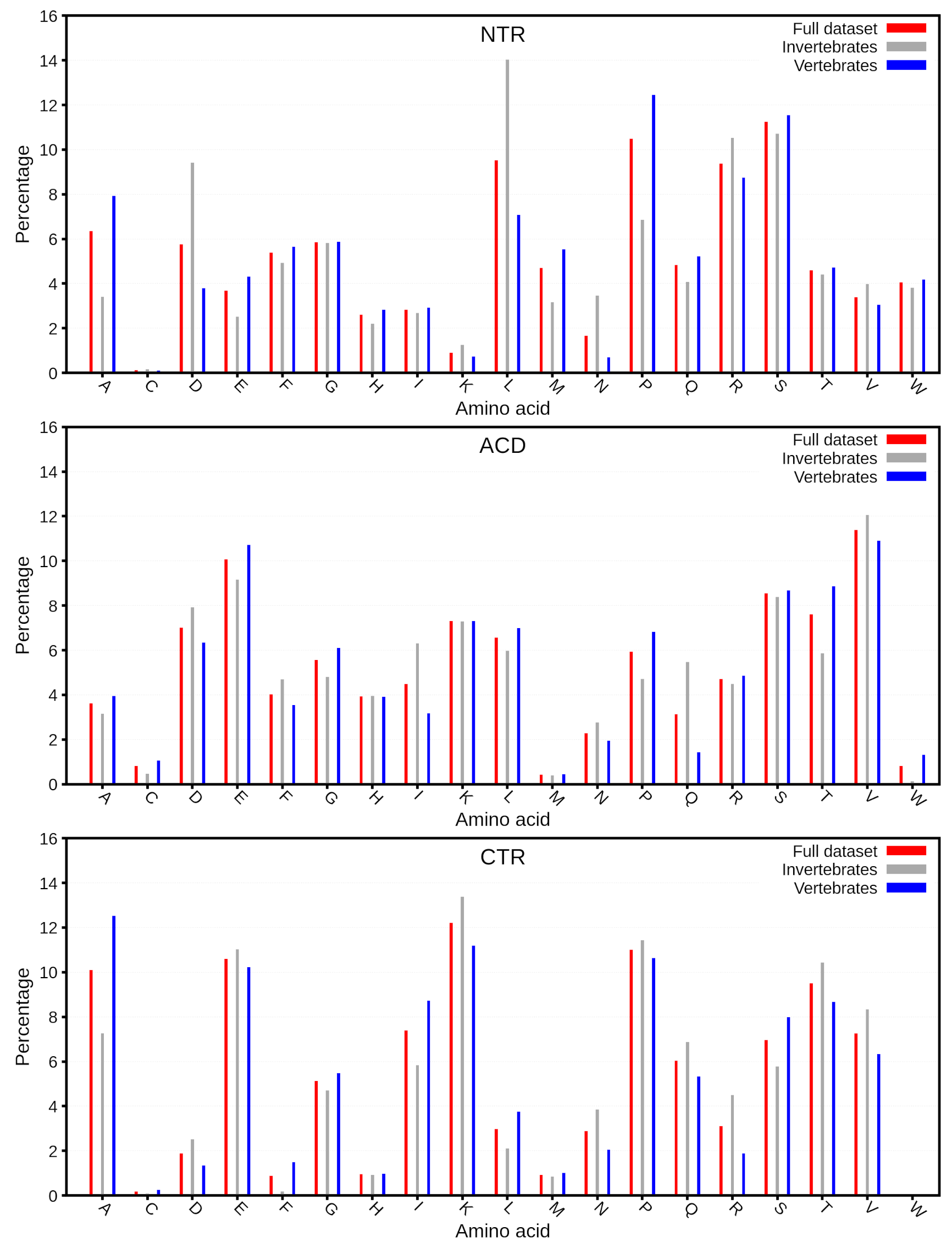

Supplement: S1 Fig — (TIF) [file pone.0321163.s001.tif]

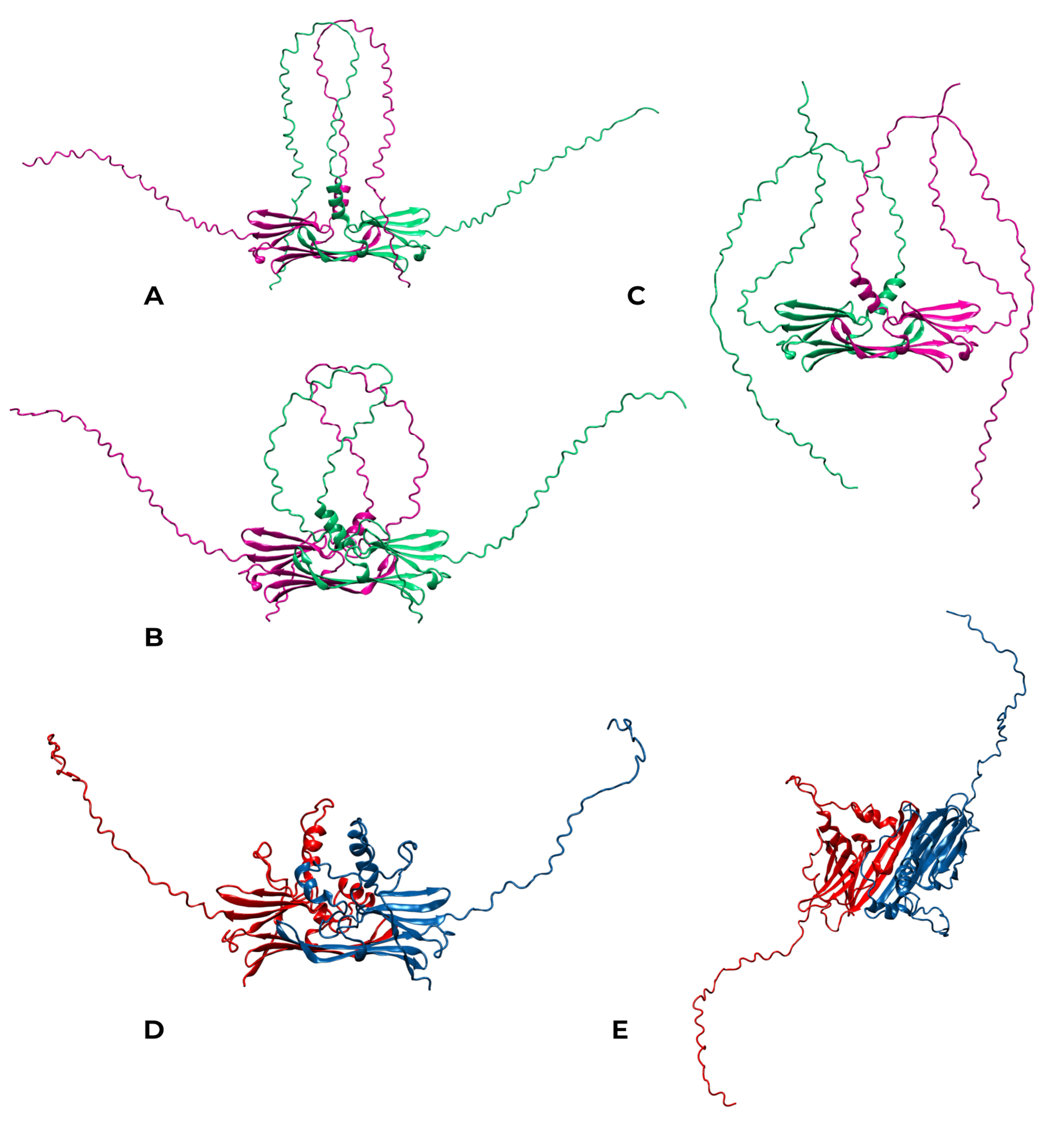

Supplement: S2 Fig — Models A and B depict interchain and intrachain interactions between the NTR and the ACD, with the CTR remaining in solution. Model C shows a conformation where neither the NTR nor the CTR interacts with the ACD. This modeling aimed to determine whether interactions between the 6VPFSLL11 motif and the ACD could be captured, regardless of the specific conformations adopted by the rest of the NTR. Models D and E display two perspectives of the same conformation from a dimer model generated using HSPB1’s phosphomimetic sequence, modeled with ColabFold [61] and employing PDB 4MJH as a template. In the highest-ranked model among the five provided by the server, the distal (which adopts a β-sheet structure upon binding to the lateral grooves), aromatic, conserved, Trp-rich, inserted, and boundary segments adopt conformations consistent with the experimental description provided for each region by Clouser et al., while the CTR remains in solution. (TIF) [file pone.0321163.s002.tif]

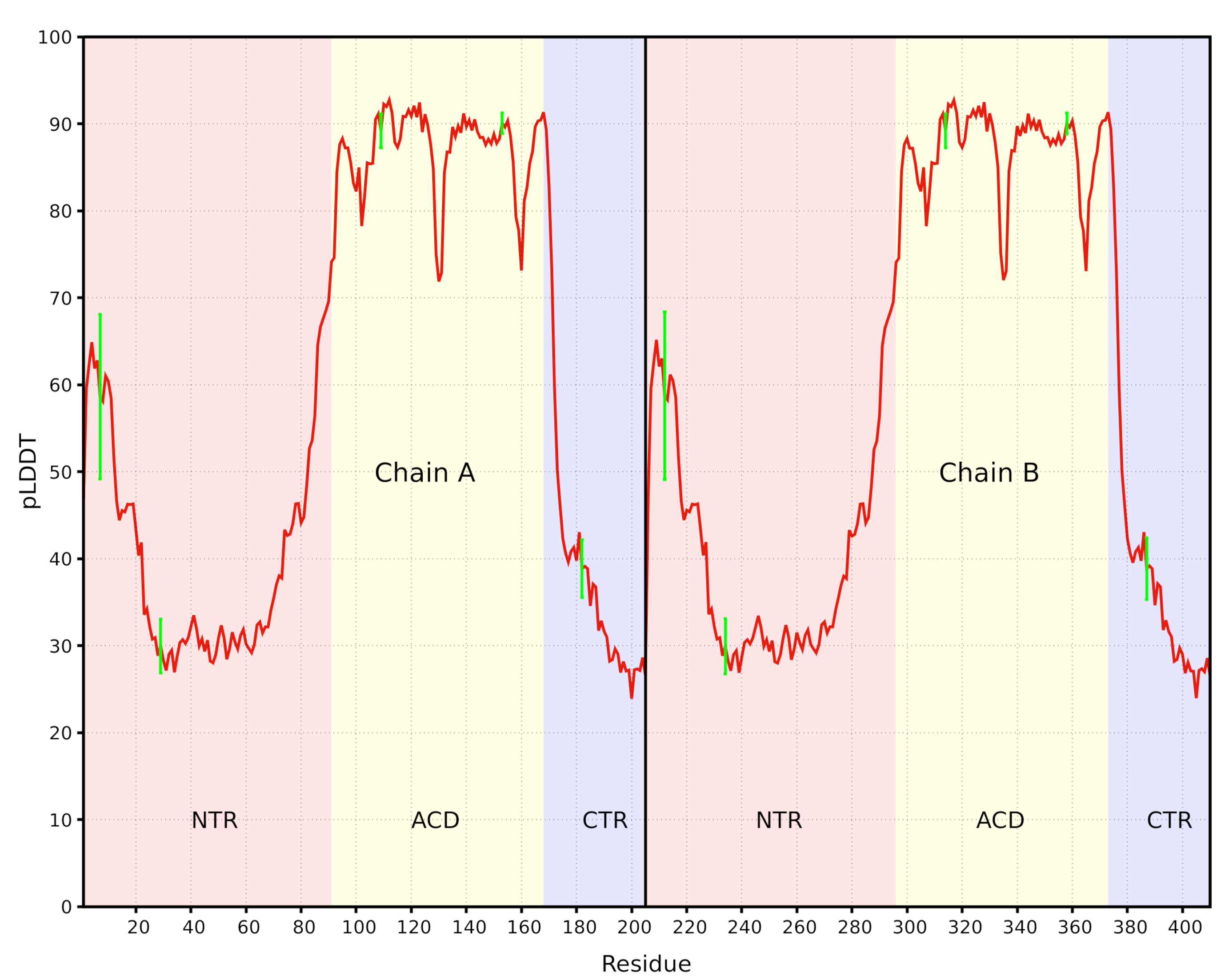

Supplement: S3 Fig — Residues 1 to 205 correspond to Chain A, and residues 206 to 410 correspond to Chain B. Background colors highlight the residue ranges for the NTR, ACD, and CTR. Green error bars indicate the standard deviation for key residues across different regions: P7 (from the 6VPFSLL11 motif), F29 (from the conserved 26SRLFDQXFG34 motif), L109 (β4 strand), V153 (β8 strand), and P182 (from the 179ITIPV183 motif). (TIF) [file pone.0321163.s003.tif]
