# Supplementary material for: Coevolution in human small Heat Shock Protein 1 is promoted by interactions between the Alpha-Crystallin domain and the disordered regions
Source: PLoS One. 2025 May 5;20(5):e0321163. doi: 10.1371/journal.pone.0321163 (PMC12052118; doi:10.1371/journal.pone.0321163)
Supplement: S1Table — For the motifs located in disordered regions, the three most frequent alternative motifs in each alignment are indicated. Only the percentage of the predominant amino acids (up to three) is shown in each position. (DOCX) [file pone.0321163.s004.docx]

| **Amino acid composition per position of the interacting motifs** | | | | |
| --- | --- | --- | --- | --- |
| Dataset | _6_VPFSLL_11_ | | _179_ITIPV_183_ | |
|  | % Composition | Most frequent alternative sequences | % Composition | Most frequent alternative sequences |
| Vertebrates | V V=55.6, I=44.4  P P=100.0  F F=98.6, L=0.7, T=0.4  T T=57.8, S=40.7, A=1.1  L L=53.8, F=37.8, M=5.1  L L=85.1, M=5.5, Q=4.0 | VPFSLL: 27.6%  VPFTFL: 21.5%  IPFTLL: 13.8% | I I=66.6, T=24.4 V=6.2  T T=82.9, S=5.8, N=5.5  I I=98.9, F=0.4, Q=0.4  P P=99.3, R=0.3, E=0.4  V V=96.4, I=2.6, S=0.7 | ITIPV: 56.0% TTIPV: 20.4%  INIPV: 4.4% |
| Invertebrates | V V=66.8, L=22.6, I=5.5  P P=100.0  L L=61.3 F=15.1, M=11.6,  L L=38.2 M=28.6, V=13.1,  F F=73.9, L=13.1, Y=6.0  - -=98.5, P=1.0, G=0.5 | VPLMF: 19.6% VPLLF: 14.1%  VPLVF: 6.0% | V V=72.4, I=27.1, -=0.5  P P=70.9, Q=9.6, S=4.5  I I=88.4, V=10.6, L=0.5 | VPI: 53.8% IPI: 12.1%  VQI: 8.5% |
| Full dataset | V V=60.3, I=28.1, L=9.5  P P=100.0  F F=63.5, L=26.2, M=4.9  T T=34.2, S=23.8, L=16.0  F F=53.0, L=36.7, M=3.2  L L=49.4, R=25.1, D=4.2 | VPFSLL: 16.0%  VPFTFL: 12.5%  VPLMFR: 8.2% | I I=68.8, V=30.4, L=0.2  P P=87.6 Q=4.0, N=1.9  V V=60.3, I=38.6, L=0.2 | IPV: 56.3%  VPI: 22.3%  IPI: 6.5% |
| **Amino acid composition of the lateral grooves** | | | | |
| Dataset | _109_LTVKT_113_ (β4 strand) | | _153_VSSSL_157_ (β8 strand) | |
|  | % Composition | | % Composition | |
| Vertebrates | L L=98.6, I=1.5  V V=52.7, T=38.6, M=3.6  V V=98.6, I=1.5  K K=98.9, R=1.1  T T=97.8, M=2.2 | | V V=91.3, I=8.4  S S=34.9, T=32.7, R=17.1  S S=98.6, P=0.7, A=0.4  S S=89.8, T=5.1, A=2.9  L L=99.6, A=0.4 | |
| Invertebrates | I I=94.0, L=4.0, V=2.0  T T=60.3, S=21.6 N=7.5  V V=99.0, I=1.0  K K=97.5, R=2.5  T T=62.3, V=25.1, I=7.0 | | V V=77.9, I=18.1 L=4.0  T T=37.7, V=25.1 E=16.6  S S=98.5, C=1.5  S S=50.8, T=19.6, R=12.6  L L=99.5, I=0.5 | |
| Full dataset | L L=58.9, I=40.3, V=0.8  T T=47.7, V=31.2, S=9.7  V V=98.7, I=1.3  K K=98.3 R=1.7  T T=82.9, V=10.6, I=3.0 | | V V=85.7, I=12.5, L=1.7  T T=34.8, S=23.2, V=12.9  S S=98.5, C=0.8, P=0.4  S S=73.4, T=11.2, R=5.3  L L=99.6, I=0.2, A=0.2 | |
