# Supplementary material for: Coevolution in human small Heat Shock Protein 1 is promoted by interactions between the Alpha-Crystallin domain and the disordered regions
Source: PLoS One. 2025 May 5;20(5):e0321163. doi: 10.1371/journal.pone.0321163 (PMC12052118; doi:10.1371/journal.pone.0321163)
Supplement: S2 Table — Values represent the proportion of interactions between and within each region relative to the total mapped interactions. (DOCX) [file pone.0321163.s005.docx]

| **Intrachain interactions (total number of mapped interactions = 2811)** | | | |
| --- | --- | --- | --- |
|  | NTR | ACD | CTR |
| NTR | 34.2% | 4.4% | 2.5% |
| ACD | 4.4% | 48.4% | 3.7% |
| CTR | 2.5% | 3.7% | 6.8% |
| **Interchain interactions (total number of mapped interactions = 830)** | | | |
|  | NTR | ACD | CTR |
| NTR | 57.5% | 0.0% | 9.1% |
| ACD | 0.0% | 22.9% | 10.5% |
| CTR | 9.1% | 10.5% | 0.0% |
